# Supplementary material for: Percentage of Asymptomatic Infections among SARS-CoV-2 Omicron Variant-Positive Individuals: A Systematic Review and Meta-Analysis
Source: Vaccines (Basel). 2022 Jun 30;10(7):1049. doi: 10.3390/vaccines10071049 (PMC9321237; doi:10.3390/vaccines10071049)
Supplement: Supplementary file 1 [file vaccines-10-01049-s001.zip › vaccines-1766815-supplementary.pdf]

Supplemental material

Text S1: Search strategy

Pubmed: Advance Search :

1 ("COVID-19"[Mesh]) OR ("SARS-CoV-2"[Mesh])

2 ((((Severe Acute Respiratory Syndrome Coronavirus 2[Title/Abstract]) OR (sars-cov*[Title/Abstract])) OR (SARS Coronavirus*[Title/Abstract])) OR (covid*[Title/Abstract])) OR (omicron[Title/Abstract])

3 **1** OR **2**

4 asymptomatic infections[MeSH Terms]

5 (asymp*[Title/Abstract]) OR (pre-symp*[Title/Abstract]) OR (presymp*[Title/Abstract]) OR (preclinical[Title/Abstract]) OR (pre-clinical[Title/Abstract]) OR (subclinical[Title/Abstract]) OR (sub-clinical[Title/Abstract]) OR (without symptoms[Title/Abstract]) OR (no symptoms[Title/Abstract]) OR (free of symptoms[Title/Abstract]) OR (non-symp*[Title/Abstract]) OR (nonsymp*[Title/Abstract]) OR (symptom- free[Title/Abstract]) OR (symptom free[Title/Abstract])

6 **4** OR **5**

7 ("2021/11/26"[Date - Completion] : "2022/04/13"[Date - Completion])

8 **3** AND **6** AND **7**

Embase: Advance Search :

#1 'severe acute respiratory syndrome coronavirus 2'/exp

#2 'coronavirus disease 2019'/exp

#3 **#1** OR **#2**

#4 'severe acute respiratory syndrome coronavirus 2':ab,ti OR 'sars cov*':ab,ti OR 'sars coronavirus*':ab,ti OR covid*:ab,ti OR omicron:ab,ti

#5 **#3** OR **#4**

#6 'asymptomatic infection'/exp

#7 'asymptomatic coronavirus disease 2019'/exp

#8 **#6** OR **#7**

#9 asymp*:ab,ti OR 'pre symp*':ab,ti OR presymp*:ab,ti OR preclinical:ab,ti OR 'pre clinical':ab,ti OR subclinical:ab,ti OR 'sub clinical':ab,ti OR 'without symptoms':ab,ti OR 'no symptoms':ab,ti OR 'free of symptoms':ab,ti OR nonsymp*:ab,ti OR 'non symp*':ab,ti OR 'symptom free':ab,ti OR symptomfree:ab,ti

#10 **#8** OR **#9**

#11 [26-11-2021]/sd NOT [13-04-2022]/sd

#12 **#5 AND #10 AND #11**

Web of Science: Advance Search :

1 TS=(SARS-CoV* OR covid* OR Severe acute respiratory syndrome coronavirus 2 OR coronavirus disease 2019 OR SARS Coronavirus* OR omicron)

2 TS=(asymp* OR pre-symp* OR presymp* OR preclinical OR pre-clinical OR subclinical OR sub-clinical OR without symptoms OR no symptoms OR free of symptoms OR non-symp* OR nonsymp* OR symptom-free OR symptom free)

3 2021-11-26 to 2022-04-13 (publish date)

4 **1** AND **2** AND **3**

Table S1: Study Quality Assessments

Cohort studies

1.1 Is the cohort representative of the target population?	1.2 Is there likely selection bias?	2.1 Was there a clear definition for an asymptomatic case?	2.2 Is there likely reporting bias?	3.1 Were objective, standard criteria used for diagnosis of the condition?	3.2 Was symptom development assessed in asymptomatic subjects?	3.3 Was follow-up long enough for symptoms to occur? (14 days on average)	3.4 Were symptoms assessed in a systematic and reliable way?	3.5 Adequacy of follow up of cohorts (lost to follow up)	3.6 Is there likely detection bias?

Grading scale: If question 1.2 is answered “yes” then the study is automatically low quality. If one question assessing bias (2.2 or 3.6) is answered "yes" then study is moderate quality. If both questions assessing bias (2.2 and 3.6) are answered "yes" then the study is low quality.

Cross-sectional studies

1.1 Were the criteria for inclusion in the sample clearly defined?	1.2 Was an appropriate method of sampling used? Random, complete, other.	1.3 Participation rate	1.4 Were the study subjects representative of the target population?	1.5 Is there likely to be selection bias?	2.1 Was there a clear definition for an asymptomatic case?	2.2 Were the study subjects and the setting described in detail?	2.3 Is there likely to be reporting bias?	3.1 Were objective, standard criteria used for diagnosis of the condition?	3.2 Was symptom assessment carried out in a standard objective way?	3.3 Is there likely detection bias?

Grading scale: If questions 1.2 is not a random sample, or if question 1.3 has a lower than 70% participation rate, then the study is automatically low quality. If one question assessing bias (1.5, 2.3 or 3.3) is answered "yes" then study is moderate quality. If two or more questions assessing bias (1.5, 2.3 or 3.3) are answered "yes" then the study is low quality.

Subgroup	No. of studies	Percentage (95%CI)	I^2 value, %	P value	Weight,%
**Development level**					
Developed	5	28.66 (14.31–43.01)	97.6	<0.001	63.1
Developing	3	38.93 (0.00–81.43)	98.3	<0.001	36.9
**Pubilication year**					
2021	1	23.57 (20.35–26.80)	NA	NA	12.6
2022	7	33.72 (20.55–46.90)	98.0	<0.001	87.4
**Number of Omicron cases**					
<500	3	46.03 (22.19–69.87)	96.8	<0.001	36.4
≥500	5	24.60 (14.42–34.78)	92.1	<0.001	63.6
**Study design**					
Cohort study	3	34.12 (7.36–60.88)	96.8	<0.001	37.4
Cross-sectional study	5	31.44 (11.21–51.66)	98.2	<0.001	62.6
**Study quality**					
Low	3	24.23 (0.00–52.77)	98.3	<0.001	38.1
Moderate	5	37.48 (20.89–54.06)	97.4	<0.001	61.9
**Transmission place**					
Community	3	37.97 (0.00–92.83)	99.1	<0.001	36.8
Others	5	29.28 (20.36–38.20)	94.1	<0.001	63.2
**Travel history**					
No	6	29.98 (18.28–41.69)	97.4	<0.001	75.4
Yes	2	40.05 (0.00–100.00)	99.0	<0.001	24.6
**Vaccine coverage**					
<80%	3	31.23 (0.00–70.20)	98.8	<0.001	42.9
≥80%	4	35.93 (12.29–59.58)	97.6	<0.001	57.1
**Male to female ratio**					
0.5 to <1.0	2	36.94 (28.54–45.35)	0.0	0.62	50.2
1.0 to 1.5	2	40.05 (0.00–100.00)	99.0	<0.001	49.8
**Median age, years**					
<20	1	43.75 (38.45–49.05)	NA	NA	24.1
20-40	3	32.59 (12.91–52.27)	95.0	0.006	75.9
**Combined percentage**	8	32.40 (20.99–43.82)	97.7	<0.001	100.0

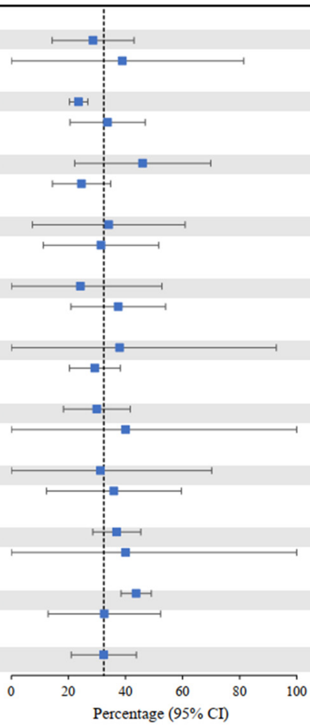

Figure S1: The Percentage of Asymptomatic Infections Among the SARS-CoV-2 Omicron variant-positive individuals by Subgroups, Using the Knapp-Hartung Adjustments
